# Analgesic effect of oral ibuprofen 400, 600, and 800 mg; paracetamol 500 and 1000 mg; and paracetamol 1000 mg plus 60 mg codeine in acute postoperative pain: a single-dose, randomized, placebo-controlled, and double-blind study

**DOI:** 10.1007/s00228-021-03231-9

**Published:** 2021-10-16

**Authors:** Gaute Lyngstad, Per Skjelbred, David M. Swanson, Lasse A. Skoglund

**Affiliations:** 1 Section of Dental Pharmacology and Pharmacotherapy, Institute of Clinical Dentistry, Faculty of Dentistry, University of Oslo, Blindern, P. O. Box 1119, N-0317 Nydalen Oslo, Norway; 2 Department of Maxillofacial Surgery, Oslo University Hospital, P. O. Box 4950, Nydalen N-0424 Oslo, Norway; 3 Oslo Centre for Biostatistics and Epidemiology, Oslo University Hospital, Blindern, P.O. Box 1122, N-0317 Oslo, Norway

**Keywords:** Ibuprofen, Paracetamol, Codeine, Postoperative pain, Third molar

## Abstract

**Purpose:**

Effect size estimates of analgesic drugs can be misleading. Ibuprofen (400 mg, 600 mg, 800 mg), paracetamol (1000 mg, 500 mg), paracetamol 1000 mg/codeine 60 mg, and placebo were investigated to establish the multidimensional pharmacodynamic profiles of each drug on acute pain with calculated effect size estimates.

**Methods:**

A randomized, double-blind, single-dose, placebo-controlled, parallel-group, single-centre, outpatient, and single-dose study used 350 patients (mean age 25 year, range 18 to 30 years) of homogenous ethnicity after third molar surgery. Primary outcome was sum pain intensity over 6 h. Secondary outcomes were time to analgesic onset, duration of analgesia, time to rescue drug intake, number of patients taking rescue drug, sum pain intensity difference, maximum pain intensity difference, time to maximum pain intensity difference, number needed to treat values, adverse effects, overall drug assessment as patient-reported outcome measure (PROM), and the effect size estimates NNT and NNTp.

**Results:**

Ibuprofen doses above 400 mg do not significantly increase analgesic effect. Paracetamol has a very flat analgesic dose–response profile. Paracetamol 1000/codeine 60 mg gives similar analgesia as ibuprofen from 400 mg, but has a shorter time to analgesic onset. Active drugs show no significant difference in maximal analgesic effect. Other secondary outcomes support these findings. The frequencies of adverse effects were low, mild to moderate in all active groups. NNT and NTTp values did not coincide well with PROMs.

**Conclusion:**

Ibuprofen doses above 400 mg for acute pain offer limited analgesic gain. Paracetamol 1000 mg/codeine 60 mg is comparable to ibuprofen doses from 400 mg. Calculated effect size estimates and PROM in our study seem not to relate well as clinical analgesic efficacy estimators.

**Trial registration:**

NCT00699114.

**Supplementary Information:**

The online version contains supplementary material available at 10.1007/s00228-021-03231-9.

## Introduction

Numbers needed to treat (NNT) are frequently used as a measure of the clinical efficacy of analgesics on acute postoperative pain [1]. Concerns have been raised over the convenience of using NNT values as non-procedure-specific evidence of pain relief as they may be misleading [2]. They may not be representative for all postoperative pain types as they integrate data from trials with confounding factors such as mix of different pain modalities, intensities [2–4], ages, and nonhomogeneous ethnical patient populations [5, 6]. NNT values represent a monodimensional effect measure, which do not take into account the complete pharmacodynamic profile of analgesics including time-related variables, and patient-reported outcome measures (PROM).

The objective of this multidimensional study was to investigate the relative clinical pharmacodynamic profiles of commonly used doses of ibuprofen, paracetamol, paracetamol with codeine, versus placebo using the well documented dental impaction model with balanced entry pain and an ethnically homogenous study population [7]. We compared these profiles with the calculated NNT values of the respective analgesics in this test model. The clinical relevance of this multidimensional study was to establish minimum drug doses with maximum benefit to avoid unnecessary overdosing or suboptimal dosing of these types of analgesics when used for postoperative pain after limited surgical interventions.

## Methods

### Design and ethical practices

This was a prospective randomized, double-blind, single-dose, placebo-controlled, parallel-group, single-centre, outpatient, and fixed-dose study. The design included a screening period (at least 14 days before surgery), surgical period, qualification period waiting for local anaesthesia to wear off (up to 6 h after surgery), and an observation period of 6 h.

The trial design and performance was approved by the following Norwegian committees: Regional committee for Ethical Medical Research (REK, South 2.2007.108, date 20.2.2007), The Norwegian Social Science Data Services (16,054, date 16.2.2007), and The Norwegian Medicines Agency, NoMA (EudraCT 2006–006,096-20, date 2.11.2006). Patients provided written informed consent prior to any screening- and study-related procedures. The patients were not informed and treated surgically by the same person, or received remunerations.

### Patients and inclusion/exclusion criteria

Eligible participants of both sexes were aged between 18 and 30 years of Norwegian Caucasian origin, referred to the Department of Maxillofacial Surgery, Oslo University Hospital, for surgical removal of impacted third molars. Patients were included if they reached “moderate” on a 5-point Likert verbal rating scale (VRS) being “no, mild, moderate, severe, or very severe pain” verified by ≥ 4 on a horizontal 11-point numerical visual analogue rating scale (NRS) running from “no pain = 0” to “worst imaginable pain = 10” within the qualification period [8]. Exclusion criteria were ASA-classification > II; ongoing drug treatment except contraceptives; use of analgesics 3 days prior to the surgery; pregnancy or planned conception; or known contraindication to NSAIDs, paracetamol, or opioids.

### Randomization and blinding

Prior to the trial, a sequentially numbered medication allocation list was made by LAS using a computer-generated system (www.randomization.com, seed number 3538). The randomized list contained seven treatment groups identified by the letters A to G, in blocks containing 14 patients. Each of the 7 trial drugs was then assigned randomly to one of the treatment groups by a person not involved in the trial. Ibumetin® (ibuprofen 200 mg, Nycomed Pharma, Norway), Pinex® (paracetamol 500 mg, Actavis, Iceland), and Pinex Forte® (paracetamol 500 mg/codeine phosphate hemihydrate 30 mg, Actavis, Iceland) were commercially purchased, processed, and identically blinded in unmarked gelatine capsules, by the Oslo University Hospital Pharmacy according to GMP-standards. Each single-trial dose contained four capsules packed in sequentially numbered envelopes to be opened by the patients. Packaging according to the randomized list was done by a person not involved in the trial. Active drug doses were ibuprofen 400 mg (IBU400), 600 mg (IBU600), and 800 mg (IBU800); paracetamol 500 (PAR500) mg and 1000 mg (PAR1000); and paracetamol 1000 mg plus codeine 60 mg (PARCOD). Capsules filled with lactose were used as placebo. All persons involved in the trial were blinded with respect to trial drug identity.

### Procedure

A standardized method of surgery [9] was used by two surgeons (GL and LAS) under local anaesthesia using lidocaine 20 mg/ml plus 12.5 μg/ml epinephrine (Xylocaine Dental Adrenalin®, Dentsply, Surrey, England). Standardized instruction on how to complete the clinical record forms (CRF) was given according to a pre-determined protocol. Patients reaching “moderate” pain on the VRS during the qualification period of 6 h self-administered the trial drug under supervision. The principal investigator (GL) was available in case of any serious adverse effects, or if any issues regarding the CRF emerged. Paracetamol 500 mg plus codeine 30 mg (Pinex Forte®) was available as rescue drugs. The patients visited the clinic 7 days after the day of surgery for postoperative control.

### Efficacy outcomes

Present pain intensity (PI) was rated on a horizontal NRS at 0 min (time of ingestion/baseline pain), 10, 20, 30, 40, 50, 60, 75, 90, and every 30 min up to 6 h post trial dose. The primary outcome measure was sum pain intensity (SPI) over 6 h after trial drug intake calculated by adding all the PI scores over 6 h. In the event of rescue drug intake, the PI score reverted to the baseline PI [10].

The secondary outcomes were time to analgesic onset defined as time between trial drug intake and first report of pain relief, duration of analgesia defined as the time between the first report of perceptible and meaningful pain relief and pain reappearing, time to rescue drug intake defined as the time from trial drug intake to intake of first rescue drug, number of patients taking rescue drug, number of rescue drug tablets, sum pain intensity difference (SPID) over 6 h, maximum pain intensity difference (MAXPID), time to MAXPID, and NNT. Pain intensity difference (PID) scores were calculated by subtracting the PI score at each time point from baseline PI, and SPID calculated equivalent to calculation of SPI. An overall assessment of the trial drugs made by the patients was used as a patient-reported outcome measure (PROM) using one each of the following alternatives: “poor”, “fair”, “good”, “very good”, or “excellent” were used as a patient-reported outcome measure [11]. Number needed to treat (NNT) and number needed to prevent the use of rescue drug (NNTp) were calculated with placebo as comparator.

### Adverse effects

The patients were instructed to report any event considered to be an AE related to the trial drugs on the questionnaires, and by interview during the follow-up visit on the 7th postoperative day. The AEs were labelled as none, mild, moderate, or severe, and the type of reported AE was recorded on the questionnaire.

### Sample size estimation

We used pooled standard deviation and effects measured in a previous trial using the same model, design, and patient population to determine sample size [12]. A sample size of 23 patients per active group would give 80% power to show a difference of at least 42% in SPI between paracetamol 1000 mg/codeine 60 mg and placebo with a two-tailed type 1 error rate of 0.05. For paracetamol 1000 mg with a SPI difference of 23%, the calculated sample size was 49. A theoretical sample size of 204 patients was necessary under the same condition to show a minimum difference of SPI of 25% between paracetamol 1000 mg and paracetamol 1000 mg/codeine 60 mg. We judged this sample size not to be relevant with respect to distinguishing clinically meaningful pain relief between active drugs within a homogenous patient population. Fifty patients were chosen for each treatment group in our study.

### Statistical methods

All data were quality-checked after trial completion, and locked for any corrections. The intention to treat (ITT) population was analyzed. All clinical endpoints were first tested for an overall effect of the trial drug groups, and pairwise comparisons between groups were done if statistical significance was achieved with respect to overall group effect. Standard descriptive statistics as mean, median, range, 95% confidence intervals (95% CI), and 1st and 3rd quartile (Q1, Q3) were used to describe the endpoints where appropriate for a useful comparison with previously published studies.

The primary variable SPI and the secondary variables SPID and duration of analgesia were analyzed with the one-way ANOVA test with the Bonferroni post hoc test. Demographic and surgical variables, baseline pain (NRS and VRS), and MAXPID, number of rescue drug tablets, were analyzed with the independent samples Kruskal–Wallis test. Gender distribution and frequency of smokers were analyzed with the Pearson chi-square test. For time-to-event outcomes (i.e. time to analgesic onset, time to MAXPID, and time to rescue drug), the log-rank Holm-Sidak test for multiple comparisons was used. PROMs were analyzed with the Joncheere-Terpstra test. The frequencies of adverse effects and frequencies used for calculating NNT and NNTp were analyzed using an equal proportion *Z*-test. Responder NNT was defined as a SPI score ≤ 50% of SPI 0–360 using baseline PI [13]. Percentages of patients taking recue drug or no rescue drug were used for NNTp calculations. All tests were 2-sided and analyzed with SPSS v. 24.0 and R version 3.5.2 [14, 15]. When multiple group analysis showed a statistical significant difference within an efficacy outcome, only the post hoc analysis *p*-values are shown. *P*-values < 0.05 were considered significant. Calculation of sample size was performed using PS: Power and Sample Size Calculations v. 2.1.30 [16].

## Results

### Participant enrolment

**Fig. 1:**
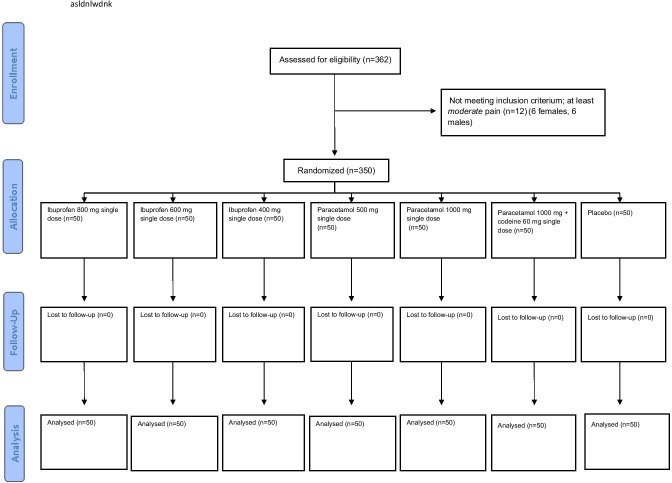
CONSORT flowchart

Of 362 patients, 350 patients fulfilled the inclusion criteria between June 2008 and June 2010, completed the trial successfully and was eligible for statistical analyses (Fig. 1). Twelve patients did not experience “moderate” pain. The study population consisted of 14.2% more females than males. Patient and surgical characteristics were adequately matched with the exception of BMI in one group. The mean and range BMI in PAR500 were slightly higher than those in IBU400, PAR1000, and PARCOD (*p* < 0.05), but within the WHO/Europe’s definition of normal BMI (18.5–24.9). There was no statistically significant difference in PI NRS between the groups when trial drugs were taken, and initial VRS was ≥ “moderate pain” for all patients (Table 1).

**Table 1:** The primary outcome, sum pain intensity (SPI), baseline pain at drug intake, and time sensitive outcomes for each trial group are shown. Present pain was scored on horizontal 11-point numerical rating scales (NRS) running from 0 (no pain) to 10 (worst imaginable pain)

	Ibuprofen 800 mg *n* = 50	Ibuprofen 600 mg *n* = 50	Ibuprofen 400 mg *n* = 50	Paracetamol 1000 mg *n* = 50	Paracetamol 500 mg *n* = 50	Paracetamol/codeine 1000 mg/60 mg *n* = 50	Placebo *n* = 50
*Baseline pain (NRS)*							
Median	5.0	5.0	5.0	5.0	5.0	5.0	5.0
(Q1, Q3)	(4.0, 6.0)	(4.0, 6.0)	(4.0, 6.0)	(4.0, 6.0)	(4.0, 6.0)	(4.0, 6.3)	(4.0, 6.0)
Mean	5.3	5.1	5.3	5.3	5.2	5.3	5.4
(95% CI)	(5.0, 5.7)	(4.8, 5.5)	(5.0, 5.7)	(4.9, 5.6)	(4.9, 5.6)	(5.0, 5.7)	(5.0, 5.9)
**Primary variable**							
*Sum pain intensity (SPI)*							
Median	45.5	45.0	60.0	60.0	69.5	54.5	90.0
(Q1, Q3)	(29.0, 60.0)	(34.0, 69.0)	(42.0, 71.0)	(52.0, 88.0)	(56.0, 83.0)	(40.0, 77.0)	(69.0, 108.0)
Mean	48.0	50.1	59.4	66.8	69.1	58.5	89.0
(95% CI)	(41.1, 54.9)	(44.1, 56.1)	(53.7, 65.2)	(59.5, 74.1)	(62.2, 76.1)	(51.4, 65.5)	(81.1, 100.0)
**Secondary variables**							
*Time to analgesic onset (min)*							
Median	32	35	36	30	30	25	365
(25, 75 quart)	(25, 44)	(25, 47)	(30, 51)	(20, 50)	(20, 40)	(15, 30)	(40, 365)
Mean	53	56	58	67	75	40	234
(95% CI)	(30, 76)	(33, 79)	(35, 81)	(38, 96)	(41, 109)	(20, 60)	(188, 280)
*Duration of analgesia (min)*							
Median	332	283	268	210	193	240	0
(25, 75 quart)	(230, 390)	(162, 341)	(190, 323)	(116, 270)	(50, 280)	(150, 315)	(0, 76)
Mean	335	288	252	204	177	234	78
(95% CI)	(274, 397)	(235, 341)	(219, 284)	(170, 238)	(141, 213)	(200, 267)	(36, 119)
*Time to rescue drug (min)*							
Median	365	365	365	315	250	365	105
(25, 75 quart)	(365, 365)	(365, 365)	(250, 365)	(210, 365)	(100, 365)	(230,365)	(60, 365)
Mean	334	328	304	278	232	298	170
(95% CI)	(311, 356)	(306, 351)	(277, 331)	(249, 307)	(197, 267)	(273, 323)	(132, 209)

### Analgesic efficacy outcome analyses

#### Primary endpoint

##### Sum pain intensity

**Fig. 2:**
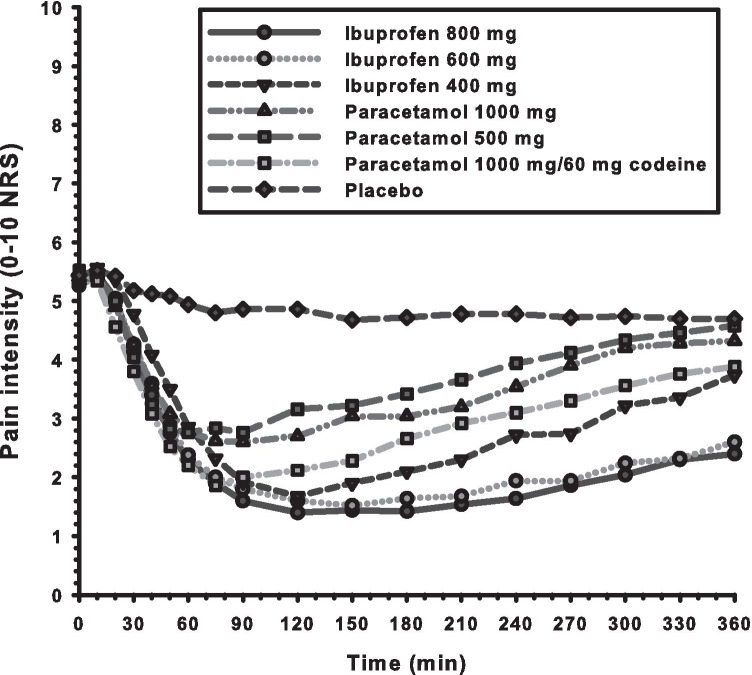
The graph shows the mean pain intensities after ibuprofen 800 mg (IBU800), 600 mg (IBU600), 400 mg (IBU400); paracetamol 1000 mg (PAR1000) and 500 mg (PAR500); paracetamol 1000 mg/codeine 60 mg (PARCOD); and placebo over the 6-h trial period. Missing data, due to intake of rescue drug by patients, are replaced with the individual baseline pain scored at trial drug intake

Post hoc comparisons between groups showed analgesic superiority for IBU800 and IBU600 over PAR1000 (*p* < 0.004 and *p* < 0.02, respectively), and over PAR500 (*p* < 0.001 and *p* < 0.003, respectively). IBU800, IBU600, IBU400, and PARCOD were not significantly different from one another, nor PAR1000 and PAR500 from one another. All active drugs were superior to placebo (all *p-*values < 0.002). SPI results are shown in Table 1, and the group PIs over the observation period are shown in Fig. 2.

#### Secondary endpoints

##### Time to analgesic onset

PARCOD showed the fastest onset of analgesia (Table 1), but was only statistically significant different to IBU400 (*p* < 0.03). There was no significant difference between the other active groups in time to analgesic onset. All active groups were superior to placebo (*p* < 0.0001).

## Duration of analgesia

IBU800 showed the longest duration of analgesia (Table 1), but was only statistically significantly different from PAR1000 (*p* < 0.001), PAR500 (*p* < 0.001), and PARCOD (*p* < 0.02). IBU600 was superior to PAR500 only (*p* < 0.007). All active drugs were superior to placebo (*p* < 0.03).

## Time to rescue drug

There was no significant difference between IBU800, IBU600, and IBU400 (Table 1), but IBU800 was superior to PAR1000 (*p* < 0.004), PAR500 (*p* < 0.0001), and PARCOD (*p* < 0.03). IBU600 was only superior to PAR1000 (*p* < 0.03), and PAR500 (*p* < 0.001). IBU400 was only superior to PAR500 (*P* = 0.04). All active treatments were superior to placebo (*p* < 0.003).

## Number of patients taking rescue drug/number needed to prevent the use of rescue drug

The number of patients taking rescue drugs and NNTp values for each group are shown in Table 2. IBU800 was different from all active groups (*p* < 0.00001) except IBU600 when analyzing the frequencies of use/non-use of rescue drug used for calculating NNTp. IBU600 was different from all the other active groups (*p* < 0.0008). IBU400 was different from PAR1000 (*p* < 0.006) and PAR500 (*p* < 0.00001). PAR500 was different from PARCOD (*p* < 0.0003) and PAR1000 (*p* < 0.015). All active groups except PAR500 was different from placebo (*p* < 0.001).

**Table 2:** PROM (patient-reported outcome measure) made on a 5-point VRS (verbal rating scale) with the alternatives “poor”, “fair”, “good”, “very good”, and “excellent”; the distribution of PROM scores; NNT (number needed to treat) values; and number of reported adverse effects with gender distribution within each treatment group are shown

	Ibuprofen 800 mg *n* = 50	Ibuprofen 600 mg *n* = 50	Ibuprofen 400 mg *n* = 50	Paracetamol 1000 mg *n* = 50	Paracetamol 500 mg *n* = 50	Paracetamol/codeine 1000 mg/60 mg *n* = 50	Placebo *n* = 50
**Secondary variables**							
*PROM (VRS)*							
Median	3	3	3	2	2	3	0
(Q1, Q3)	(2, 4)	(2, 3)	(2, 3)	(1, 3)	(1, 3)	(2, 3)	(0, 1)
Mean	3.0	2.7	2.4	1.9	1.8	2.5	0.6
(95% CI)	(2.7, 3.3)	(2.3, 3.0)	(2.1, 2.8)	(1.6, 2.2)	(1.4, 2.1)	(2.2, 2.8)	(0.4, 0.9)
*Distribution of PROM scores (%)*							
Excellent	40	22	14	8	6	18	0
Very good	32	46	40	20	26	40	6
Good	18	16	30	36	24	22	10
Fair	6	8	8	26	28	16	26
Poor	4	8	8	10	16	4	58
Sum score	100	100	100	100	100	100	100
*NNT*	1.7	1.9	4.5	7.1	8.3	3.6	n/a
*NNTp*	1.9	2.2	3.3	6.3	25	4.5	n/a
*Rescue drug*							
Patients/no tablets	9/16	12/21	20/34	27/47	33/63	24/39	35/80
*Adverse effects*							
Number of reported	1	2	2	3	4	8	2
Female/male	0/1	1/1	2/0	2/1	2/2	4/4	1/1

## Number of rescue drug tablets taken

The number of rescue drug tablets taken after IBU800 was significantly fewer (Table 2) than after PAR1000 (*p* < 0.04) and PAR500 (*p* < 0.001), but not after IBU600, or IBU400. Significantly fewer tablets were used after IBU600 than after PAR500 (*p* < 0.002). All active groups except PAR1000 and PAR500 used fewer rescue drug tablets than placebo (*p* < 0.03).

## Sum pain intensity difference

The outcome of the SPID as an analgesic response measure was identical to the SPI. IBU800 and IBU600 were only superior to PAR1000 (*p* < 0.003 and *p* < 0.04, respectively) and to PAR500 (*p* < 0.001 and *p* < 0.008, respectively). Neither IBU800, IBU600, IBU400 and PARCOD, nor PAR1000 and PAR500 were significantly different from another. All active drugs were superior to placebo (*p* < 0.004).

## Maximal pain intensity difference score

A significant difference was only found between all active drugs and placebo (*p* < 0.001).

## Time to MAXPID

IBU800 did not show a significantly different time to MAXPID compared with all other active groups. IBU400 showed significantly longer time to MAXPID than PAR1000 (*p* < 0.03) and PAR500 (*p* < 0.002). No active groups were significantly different from placebo.

## Patient-reported outcome measure

IBU800 was not significantly different from IBU600 or PARCOD (Table 2) but tended to be superior to IBU400 (*p* = 0.08). IBU800 was superior to PAR 1000 (*p* < 0.001) and to PAR500 (*p* < 0.002). IBU600 was superior to PAR 1000 (*p* < 0.006) and to PAR500 (*p* < 0.004), but not IBU400 and PARCOD. IBU400 tended to be different from PAR1000 (*p* = 0.09) and PAR500 (*p* = 0.05). PARCOD was superior to PAR500 (*p* < 0.03) and tended to be superior to PAR1000 (*p* = 0.05). All active treatments were superior to placebo (*p* < 0.001).

## Number needed to treat

Effect size estimates for active drugs compared to placebo are presented in Table 2. Analyzing the frequencies of defined responders/non-responders used for NNT calculations showed IBU800 to be superior to all active groups (*p* < 0.00001) except IBU600. IBU600 was superior to all the other active groups (*p* < 0.00001). IBU400 was superior to PAR 500 (*p* < 0.015), and tended to be superior to PAR1000 (*p* = 0.05). PARCOD was superior to PAR1000 (*p* < 0.002), and PAR500 (*p* < 0.0002). All active treatments were superior to placebo (*p* < 0.00001).

## Adverse effects

Few adverse effects, mild to moderate in nature, were reported. They were described as nausea, abdominal pressure, headache, dyspepsia with or without reflux, dizziness, detached, tired, and increased perspiration*.* PARCOD had the highest number of adverse effects, and it was significantly different from all other groups (*p* < 0.02). The adverse effects counted in the other active groups were low (Table 2). All patients fully recovered within a short time.

## Discussion

We did not find significant evidence for a clear and clinically relevant analgesic dose–response profile of ibuprofen from 400 to 800 mg, or paracetamol 500 to 1000 mg using traditional quantitative and qualitative measures of analgesic effect. There was a significant difference in analgesic efficacy between ibuprofen and paracetamol irrespective of doses. Interestingly, paracetamol 1000 mg combined with codeine 60 mg was comparable to ibuprofen in doses from and above 400 mg.

Winter et al. using non-standardized dental surgery interventions and undefined initial pain level demonstrated no difference between ibuprofen 400 mg and 800 mg [17]. Subsequently, a clear ibuprofen dose response ranging from 50 to 400 mg was found in similar pain models as ours [18, 19]. However, two dental surgery models with either higher pain level (moderate to severe) testing ibuprofen 400 mg, 600 mg, and 800 mg [20] or lower pain level (≥ 30 mm VAS) testing 200 mg, 400 mg, and 600 mg [21] also did not manage to discriminate between drug doses. Furthermore, a non-dental pain model with higher initial pain level (emergency room pain > 6 NRS) testing 400 mg, 600 mg, and 800 mg also failed to distinguish between doses [22]. Our results with an initial pain level of moderate pain add to these findings, and strongly suggest that ibuprofen reaches its analgesic ceiling at a dose of around 400 mg independent of initial pain levels or acute pain types.

Paracetamol at both doses showed analgesic inferiority compared with almost all ibuprofen doses by the analgesic measures SPI, SPID, analgesic duration, time to rescue drug intake, NNTp, and number of rescue drug tablets taken, and the PROM. Our results show only a marginal difference in clinical efficacy between paracetamol 1000 mg and 500 mg, where paracetamol 500 mg had the shortest duration of analgesia and time to intake of rescue drug. This is coincident with findings from a review using mixed pain models. A significant effect from paracetamol 1000 mg over 500 mg was shown in only four out of twelve studies, whereas nine studies showed numerical superiority of the highest paracetamol dose [23]. Our findings support the suggestion of a very flat dose–response curve for paracetamol.

The rationale for lack of correlation between analgesia and ibuprofen or paracetamol above certain doses is elusive. Studies have not been able to reveal pharmacodynamics/pharmacokinetic interrelationships for ibuprofen or paracetamol [20, 24]. Our study revealed no significant difference regarding extent of analgesic efficacy (i.e. MAXPID) between the active drugs, although the paracetamol doses had the lowest MAXPID scores. The ibuprofen groups presented the longest times to MAXPID, and ibuprofen 400 mg had significantly longer time to MAXPID than paracetamol 1000 mg and 500 mg.

One surprising finding was the apparently good analgesic effect of paracetamol 1000 mg/codeine 60 mg compared with the ibuprofen doses. The combination showed numerically the shortest time to onset and was significantly different from ibuprofen 400 mg. This observation may have favourable clinical implications if the fixed drug combination is given together with slower acting analgesics. The benefit of adding a weak opioid such as codeine to paracetamol has been a controversy and has been studied in single-dose and repeated-dose studies [12, 25, 26]. Our study demonstrated somewhat better effect of the paracetamol/codeine combination than paracetamol alone.

The predominant analgesic action of codeine is mediated through its metabolite morphine created almost exclusively by liver enzymes [27]. This enzyme is prone to a wide range of polymorphism, both inter-individual and inter-ethnic [28, 29]. The unpredictable pharmacokinetics of codeine may explain the variety of analgesic effect reported in various studies, which may have been sensitive to the selection of study populations [28]. The prevalence of the poor metabolizer phenotype is reported to be 5–11% in Caucasians [30] probably causing a limited negative effect in our study.

The frequency of adverse effects in our study was very low and consistent with the known effects of the drugs. The paracetamol/codeine combination was significantly different from the other groups with the highest number of side effects (*n* = 8). None of the reported side effects was considered serious, and all episodes were normalized within a short time. The adverse effects were too few to draw any conclusions regarding influence of gender including the paracetamol plus codeine group in which the number of reported adverse effects was equal between genders.

To the best of our knowledge, all the present analgesics have never been tested simultaneously in the same acute pain model to minimize trial confounding. We used the third molar surgery model, which is well characterized, appropriate for both low- and high-potency analgesics, and predictive for analgesic effect in other pain models [31]. Based on observed pain levels from previous trials using the same surgical technique [12, 32], we chose the minimum entry pain level “moderate” (mean score ~ 5 on a 0–10 NRS) in our study [33]. This initial pain level offered model sensitivity to distinguish between ibuprofen all doses and paracetamol/codeine versus both paracetamol doses. Our study was conducted as a single-centre study with a very homogenous population in terms of age, health status, ethnicity, and habits such as social smoking. Possible weaknesses with our pain model were the limitation of age that could limit generalization of the results, and that it was slightly dominated by females. Intra-population analysis could not show any significant gender effect on the pain data (data not shown).

Mean NNTs found in our study compared to current published (mean, 95% CI) NNT values were ibuprofen 800 mg 1.7 (1.6, 1.3–2.2), ibuprofen 600 mg 1.9 (2.7, 2.0–4.2), ibuprofen 400 mg 4.5 (2.5, 2.4–2.6), paracetamol 1000 mg 7.1 (3.6, 3.2–4.1), paracetamol 500 mg 8.3 (3.5, 2.7–4.8), and paracetamol 1000 mg/codeine 60 mg 3.6 (2.2, 1.8–2.9) [1, 34]. Our data show ibuprofen NNT values to be fairly consistent with the published NNT data, but the mixed models’ NNT values for paracetamol with or without codeine are seriously overestimated compared to those in our study. Our findings support the claim that NSAIDs may have an analgesic advantage over paracetamol in dental pain compared to major surgery [2, 35], and contradict the assertion that differences are unlikely between dental and other postsurgical pain models [36]. Our results support the concept of using procedure-specific NNTs.

The NNT values are supported by the NNTp values within the time frame limited by the defined observation period in our study. This is not unexpected since the common mathematical modelling used to create NNT values is dependent on the drug’s biological half-life, and the timing of rescue drug intake when using a defined time-dependent summed pain measure with baseline replacement data. The NNT as a single outcome estimates may give false impressions of analgesic efficacy wanted in a clinical setting. PROMs include all clinically relevant outcomes represented by the patient’s overall subjective assessment of the test drugs. This paradox is highlighted in our study by the paracetamol/codeine combination. It has a relatively high PROM score compared to ibuprofen even with the highest number of reported adverse effects, suggesting that patients may appreciate the analgesic efficacy provided.

Our study gives limited evidence of a clinically relevant analgesic effect of higher ibuprofen doses compared to ibuprofen 400 mg, and supports the concept of additional analgesic efficacy of codeine 60 mg added to paracetamol 1000 mg in this type of acute pain. This is a point, which may be considered, when using the lowest effective ibuprofen dose giving maximal analgesia for synergistic drug combinations instead of high single doses depending on the patient’s tolerability of opioid containing drugs.

## Supplementary Information

Below is the link to the electronic supplementary material.

Supplementary file1 (DOCX 15 KB)

Supplementary file2 (DOCX 15 KB)

## Data Availability

The datasets generated during and/or analyzed during the current study are available from the corresponding author on reasonable request.
