# Supplementary material for: Analgesic effect of oral ibuprofen 400, 600, and 800 mg; paracetamol 500 and 1000 mg; and paracetamol 1000 mg plus 60 mg codeine in acute postoperative pain: a single-dose, randomized, placebo-controlled, and double-blind study
Source: Eur J Clin Pharmacol. 2021 Oct 16;77(12):1843–52. doi: 10.1007/s00228-021-03231-9 (PMC8585829; doi:10.1007/s00228-021-03231-9)
Supplement: Supplementary file 1 — Supplementary file1 (DOCX 15 KB) [file 228_2021_3231_MOESM1_ESM.docx]

All Ibuprofen Ibuprofen Ibuprofen Paracetamol Paracetamol Paracetamol/codeine Placebo

patients 800 mg 600 mg 400 mg 1000 mg 500 mg 1000 mg/60 mg

n=350 n=50 n=50 n=50 n=50 n=50 n=50 n=50

*Age (years)*

Median 24 24 25 24 25 25 25 24

(Q1, Q3) (23, 26) (23, 27) (23, 26) (22, 26) (23, 27) (23, 26) (23, 26) (22, 26)

Mean 25 25 25 24 25 25 25 24

(Range) (18-30) (18-30) (19-30) (18-29) (19-29) (18-30) (18-30) (19-30)

*BMI*

Mean 22.8 23.2 22.6 22.2 22.4 24.1 22.1 23.2

(95% CI) (22.5, 23.1) (22.4, 23.9) (21.9, 23.4) (21.6, 22.8) (21.6, 23.2) (23.2, 24.9) (21.4, 22.8) (22.3, 24.1)

*Females/Males (%)* 57.1/42.9 52.0/48.0 62.0/38.0 52.0/48.0 60.0/40.0 56.0/44.0 54.0/46.0 64.0/36.0

*Smokers (%)* 10.6 10.0 6.0 14.0 8.0 8.0 18.0 10.0

*Volume local anaesthesia (ml)*

Median 3.6 3.6 4.5 3.6 3.6 3.6 3.6 3.6

(Q1, Q3) (3.2, 5.4) (3.2, 4.5) (3.6, 4.5) (3.2, 4.5) (3.2, 5.4) (3.2, 4.5) (3.6, 5.4) (3.2, 5.4)

Mean 4.2 4.0 4.6 4.0 4.2 4.0 4.4 4.2

(95 % CI) (4.1, 4.3) (3.7, 4.4) (4.2, 5.0) (3.6, 4.3) (3.9, 4.5) (3.7, 4.3) (4.1, 4.8) (3.9, 4.5)

*Duration of surgery (min)*

Median 20 20 20 15 20 20 15 17

(Q1, Q3) (15, 25) (15, 25) (15, 30) (15, 20) (15, 20) (15, 25) (15, 25) (15, 30)

Mean 20 20 22 20 20 20 20 21

(95 % CI) (19, 21) (17, 23) (20, 25) (17, 23) (17, 22) (18, 23) (17, 23) (18, 24)
