# Supplementary material for: Analgesic effect of oral ibuprofen 400, 600, and 800 mg; paracetamol 500 and 1000 mg; and paracetamol 1000 mg plus 60 mg codeine in acute postoperative pain: a single-dose, randomized, placebo-controlled, and double-blind study
Source: Eur J Clin Pharmacol. 2021 Oct 16;77(12):1843–52. doi: 10.1007/s00228-021-03231-9 (PMC8585829; doi:10.1007/s00228-021-03231-9)
Supplement: Supplementary file 2 — Supplementary file2 (DOCX 15 KB) [file 228_2021_3231_MOESM2_ESM.docx]

Ibuprofen Ibuprofen Ibuprofen Paracetamol Paracetamol Paracetamol/codeine Placebo

800 mg 600 mg 400 mg 1000 mg 500 mg 1000 mg/60 mg

n=50 n=50 n=50 n=50 n=50 n=50 n=50

**Secondary variables**

*Sum pain intensity difference (SPID)*

Median 50.5 44.5 37.5 22.0 24.5 36.0 4.0

(Q1, Q3) (34.0, 63.0) (29.0, 61.0) (19.0, 53.0) (16.0, 44.0) (5.0, 43.0) (27.0, 50.0) (-1.0, 15.0)

Mean 48.1 44.6 36.0 29.3 27.3 40.9 8.9

(95% CI) (41.0, 55.3) (37.8, 51.4) (29.0, 43.0) (22.8, 35.8) (20.6, 34.1) (33.9, 47.9) (2.7, 15.0)

*Time to MaxPID (min)*

Median 75 75 90 60 50 60 50

(25, 75 quart) (50, 120) (50, 120) (75, 120) (50, 90) (40, 75) (40, 75) (30, 150)

Mean 85 102 114 75 66 82 111

(95 % CI) (68, 103) (80, 124) (94, 135) (61, 88) (48, 85) (64, 99) (78, 144)

*MaxPID*

Median 4.0 4.0 4.0 3.0 3.0 4.0 1.0

(25, 75 quart) (3.0, 6.0) (4.0, 5.0) (3.0, 5.0) (2.0, 4.0) (1.0, 5.0) (3.0, 5.0) (1.0, 2.0)

Mean 4.2 3.9 3.7 3.1 3.2 3.9 0.7

(95 % CI) (3.6, 4.8) (3.3, 4.5) (3.0, 4.5) (2.4, 3.7) (2.5, 3.8) (3.4, 4.5) (0.0, 1.5)
